# Investigation of PLGA nanoparticles in conjunction with nuclear localization sequence for enhanced delivery of antimiR phosphorothioates in cancer cells in vitro

**DOI:** 10.1186/s12951-019-0490-2

**Published:** 2019-04-22

**Authors:** Shipra Malik, Raman Bahal

**Affiliations:** Department of Pharmaceutical Sciences, University of Connecticut, 69 North Eagleville Road, Unit 3092, Storrs, CT 06269-3092 USA

**Keywords:** NLS, antimiR, PLGA, Phosphorothioates, MicroRNA

## Abstract

Numerous first generation phosphorothioates (PS) and their derivatives have shown promise targeting mRNA for therapeutic applications and also gained market approval for their use as a drug. However, PS have not been explored for targeting microRNAs (miRNAs or miRs). In particular, efficient delivery remains a critical cog in PS-based antimiR applications. In this study, we tested and characterized a series of poly-lactic-co-glycolic-acid (PLGA) polymers of different molecular weights that can encapsulate the optimum amount of antimiR-155 PS with uniform morphology and surface charge density. We found that nuclear localization sequence substantially increases loading of antimiR-155 PS in PLGA nanoparticles. Further, in a battery of cell culture studies, we confirmed that PLGA nanoparticles encapsulated nuclear localization sequence/antimiR-155 PS combination undergoes significant intracellular delivery and results in reduced expression of miR-155. In conclusion, we successfully demonstrate the feasibility and promise of optimized PLGA nanoparticles based PS delivery in combination with nuclear localization sequence for antimiRs based therapeutics.

**Electronic supplementary material:**

The online version of this article (10.1186/s12951-019-0490-2) contains supplementary material, which is available to authorized users.

## Background

MicroRNAs (miRNAs or miRs) are 20–23 nt long non-coding RNAs (ncRNAs) that target 3′ UTR (untranslated regions) of mRNA sites and control the post-transcriptional events [1]. It has been confirmed that miRNAs are key regulators of cellular processes including post-transcriptional modifications, signal transduction, differentiation, apoptosis, as well as proliferation [2, 3]. Hence, role of miRNAs has been well established in numerous pathologies; inflammatory and autoimmune diseases, metabolic and neurological disorders, just to name a few. In particular, miRNAs are pivotal for development and progression of different cancers. There is abundant evidence that overexpression of certain miRs (also called oncomiRs) results in different types of cancers. Hence, antisense-based synthetic nucleic acid oligonucleotides that can sequence specifically bind to miRNAs via Watson–Crick base pairing rules, and inhibit their expression, present a new avenue for cancer therapy. However, in past, several classes of synthetic antisense oligonucleotides have been successfully used to target messenger RNAs (mRNAs) instead of miRNA to control gene expression and regulation [4]. Excitingly, a few antisense-based drugs have received FDA approval for treating viral [5] as well as metabolic disorders [6]. In general, antisense oligonucleotides inhibit gene expression either by (a) sterically blocking mRNA translation or (b) by inducing mRNA degradation by ribonuclease H (RNase H) [7]. Additionally, it has been established that RNase-based cleavage is more effective in inhibiting RNA [8]. Among different classes of antisense-based oligonucleotides, phosphorothioates (PS) have shown considerable promise. PS belong to first generation oligonucleotides where non-bridging phosphate oxygen atom is replaced by a sulfur atom [9]. The aforementioned chemical modification improves resistance to enzymatic degradation, elicits RNase H-mediated cleavage of the target RNA, and increases affinity for plasma proteins that subsequently increase their bioavailability.

PS remain widely used in antisense therapeutics targeting mRNA, however, few studies have highlighted the importance of PS as promising antimiR agent [10]. A clinical trial is ongoing with PS and locked nucleic acid (LNA)-based antimiR-122 drug, miravirsen that showed long-lasting suppression of viral load in hepatitis C virus (HCV)-infected patients [11]. Additionally, some studies have demonstrated that unconjugated, saline formulated PS-based oligonucleotides can be used for mRNA as well as miRNA silencing [12]. However, the main hurdle towards translational success and broader application of PS-based antimiR therapeutics is their delivery. Though some progress has been made using cationic complexes as well as charge neutral liposomes, but still stability of those formulations possess enormous challenge [13, 14]. In addition, interaction of cationic complexes with blood components leads to serum sensitivity as well as cytotoxicity [15]. Hence, development of PS-based therapeutic modalities can be further expanded, and acute and sub chronic toxicities can be reduced by utilizing effective delivery strategies. On this front, nanoscale delivery systems represent a promising platform for antimiR therapy, a key benefit being their amenability to improvements that can overcome cellular and physiological delivery barriers.

Numerous studies have been reported on FDA approved poly-lactic-co-glycolic-acid (PLGA) polymers for delivery of plasmid DNA [16], siRNA [17] and peptide nucleic acid (PNAs) [18–21]. In prior reports it has been found that PLGA nanoparticles (NPs) coated with a cell penetrating peptide can deliver PNAs-based antimiRs in vitro and in vivo [22]. However, PLGA NPs have not been explored to deliver PS-based antimiR oligonucleotides. Herein, we describe development and optimization of PLGA nanoparticles (NPs) for delivery of PS-based antimiR oligonucleotides. For proof of concept, we used antimiR-155 PS (PS-155) targeting miR-155. miR-155 in particular is one of the most salient miRNAs and is implicated in cancerous pathways including several lymphomas, such as diffuse large B cell lymphomas, Hodgkin lymphomas and subsets of Burkitt lymphomas, and ectopic expression of miR-155 in a transgenic mouse model leads to B-cell malignancy [3]. Our studies show that optimized PLGA NPs encapsulating PS-155 have superior payload. In addition, we also noted that novel nuclear localization sequence (NLS)-based counter-ions increase the loading of PS antimiRs into optimized PLGA NPs and hence increase their antimiR efficacy with favorable safety profiles. To the best of our knowledge, this is the first report exploiting the potential of PLGA NPs and NLS for improved delivery and efficacy of PS based antimiRs.

## Results

In prior studies, it has been demonstrated that ester terminated PLGA NPs (containing equal ratios of poly-lactic acid and poly-glycolic acid, 50:50) can effectively deliver antimiR PNAs ex vivo and in vivo [23, 24]. In this study, we sought out to develop NP formulations that can encapsulate and deliver optimum amount of PS-155.

**Fig. 1:**
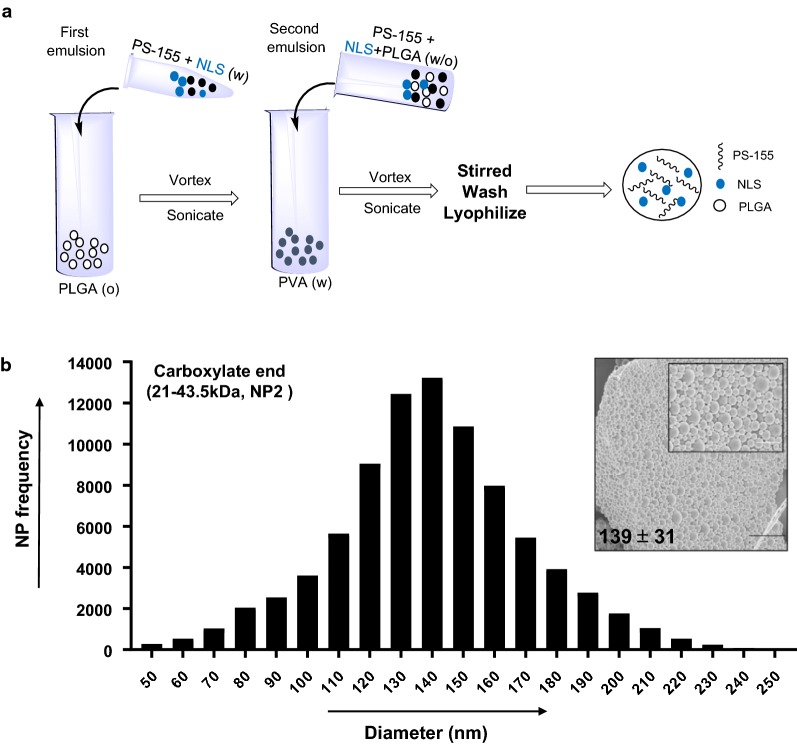
Formulation and size distribution of PLGA nanoparticles containing PS-155 and nuclear localization sequence (NLS). **a** PLGA nanoparticles were formulated using PLGA double emulsion/solvent evaporation technique. Particles were either encapsulated with PS-155 alone or co-encapsulated with NLS and PS-155. For imaging studies, TAMRA dye conjugated PS-155 were used. **b** Representative size distribution and scanning electron micrographs (SEM) (inset) of PLGA nanoparticles containing PS-155. Bar represents 1 μm. Average particle diameter and SD are given for nanoparticle batch

Initially, we tested a series of ester and acid terminated PLGA polymers (50:50) of different molecular weights (6.7–91.6 kDa) to identify the best polymer that can load optimum amount of PS-155 into NPs (Table 1). PLGA NPs were formulated using a double emulsion solvent evaporation technique (Fig. 1a). We attempted to formulate PLGA NPs encapsulating PS-155 with diameters between 100 and 150 nm to achieve efficient cellular uptake. Most notably, during NP formulations, solvent evaporation was continued overnight to ensure complete evaporation of dichloromethane (DCM). In addition, NPs were centrifuged at 9500 rpm, as opposed to 16,000 rpm, because at higher speeds, particles tend to fuse together.

**Table 1:** Characterization of different molecular weight PLGA nanoparticle (NP) preparations containing PS-155 for mean size (DLS diameter), surface charge (zeta potential) and polydispersity index (PDI)

PLGA polymer (50:50)	Formulations	Inherent viscosity (dL/g)	PLGA mol. wt. (kDa)	Zeta potential (mv ± SD)	Hydrodynamic diameter (DLS) (nm ± SD)	PDI
Acid terminated	NP-1	0.15–0.25	6.7–12.9	− 24.5 ± 1.2	280 ± 80	0.13 ± 0.06
	NP-2	0.55–0.75	21.0–43.5	− 26.5 ± 2.0	376 ± 15	0.16 ± 0.05
Ester terminated	NP-3	0.15–0.25	6.70–12.9	− 20.2 ± 4.8	358 ± 70	0.26 ± 0.09
	NP-4	0.26–0.54	12.9–21.0	− 22.4 ± 4.8	351 ± 70	0.17 ± 0.08
	NP-5	0.65	43.5	− 23.3 ± 4.6	430 ± 137	0.19 ± 0.13
	NP-6	0.95–1.2	73.7–91.6	− 18.3 ± 2.3	536 ± 32	0.16 ± 0.008

### Determination of size and surface charge of PS-155 loaded PLGA NPs

Next, we performed scanning electron microscopy (SEM) studies to determine the morphology and size distribution of PLGA NPs. Ester terminated PLGA NPs were highly fused together when viewed under SEM (Additional file 1: Fig S1) and ranged in sizes between 100 and 300 nm. Acid terminated PLGA NPs (NP2, 21–43.5 kDa) (Table 1) demonstrated uniform size (~ 139 nm) and morphology (Fig. 1b). We also assessed the hydrodynamic diameter via dynamic light scattering (DLS) studies. All NPs showed DLS diameters between 300 and 600 nm (Table 1). Additionally, zeta potential measurements were performed to determine the surface charge on the particles. All formulated PLGA NPs revealed zeta potential between − 30 and − 20 mV (Table 1).

### Loading and controlled release of PS-155 from NPs

**Fig. 2:**
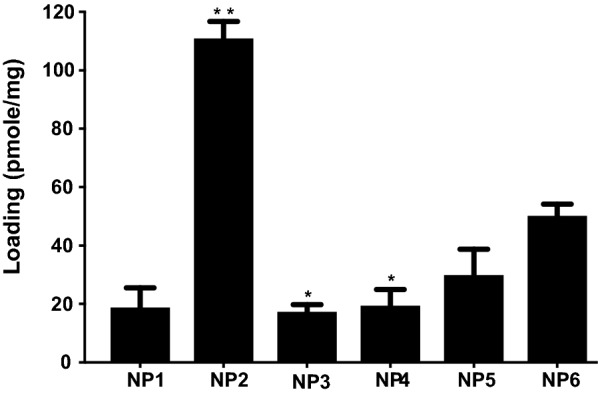
Nucleic acid loading analysis results. Loading of antimiR PS-155 nucleic acids in different grade PLGA nanoparticles. See Table 1 for description of NP1-NP6. Data are shown as mean ± standard error. (SE), n = 3. *p < 0.05)

**Fig. 3:**
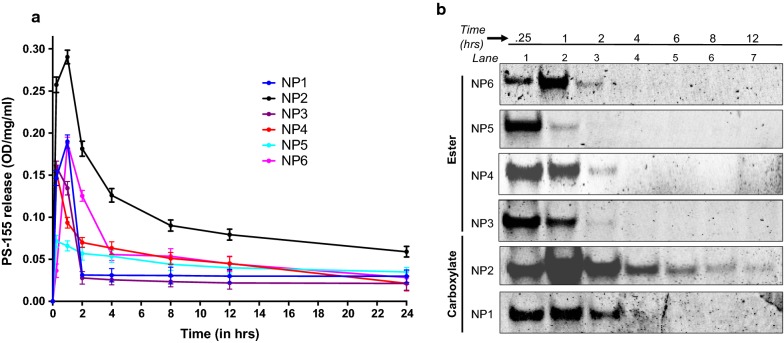
Nucleic acid release profile studies. **a** Release profile data of antimiR-155 PS nucleic acid from PLGA NPs at indicated time points in a graph. All data were collected in triplicate (n = 3) and error reported as mean ± standard error (SE). **b** Electrophoretic mobility gel shift assay to quantify the release of antimiR-155 PS from PLGA nanoparticles at indicated time points. The samples were separated on 10% nondenaturing polyacrylamide gel and stained with SYBR-Gold

Loading of PS-155 into PLGA NPs was determined by organic solvent extraction followed by assessing nucleic acid absorbance at 260 nm. Acid terminated PLGA NPs (NP2, 21–43.5 kDa) exhibited three to five folds higher loading of PS-155 as compared to other grades of PLGA polymer (Fig. 2). Further, kinetics of PS-155 release from NPs were determined by incubating NPs in phosphate buffered saline (PBS) at physiological temperature at a given time interval. Most of the particles showed burst release of PS-155 within 2 h. However, NP2 released 80% of PS-155 within first 12 h followed by tapered slow release after 24 h (Fig. 3a). Overall, we noticed increased release of PS-155 for NP2 as compared to other grade polymers which could be attributed to increased loading of PS-155 into PLGA NPs. Additionally, we optimized polyacrylamide based electrophoretic gel shift assays to quantify the release of PS-155 from different PLGA NPs (Fig. 3b). Released PS-155 was quantified by SYBR Gold staining. Gel shift results also validate our release profile results and indicate that NP2 showed sustained release of PS-155 for 12 h, whereas other grade PLGA NPs lead to burst release of PS-155 within 1 h. Overall, our NP characterization results signify the superiority of the NP2 for improved loading and release of PS-155 as compared to other formulations. Hence, we employed NP2 loaded with PS-155 for subsequent studies.

### Exploring different counter ions to increase the loading of PS-155 onto PLGA NP2

Both PS-155 and NP2 are negatively charged. So, electrostatic repulsions could lead to modest loading of PS-155 into PLGA NPs. One possible strategy to increase the payload into NP2 is by using different positively charged counter-ions in the encapsulant. Few reports have exploited the potential of positively charged counter-ions like spermidine to increase the loading of plasmid DNA or siRNA into PLGA NPs [25]. Counter-ions presumably decrease or mask the electrostatic repulsion between negatively charged cargo and PLGA polymer. We sought out to further increase the loading of PS-155 into NP2 by using different counter-ions. We investigated five positively charged excipients as counter ions: (a) Nuclear localization sequence (NLS) (b) Spermidine (c) Histidine (d) Lysine and (e) Arginine amino acids to enhance the payload of PS-155 in NP2.

**Fig. 4:**
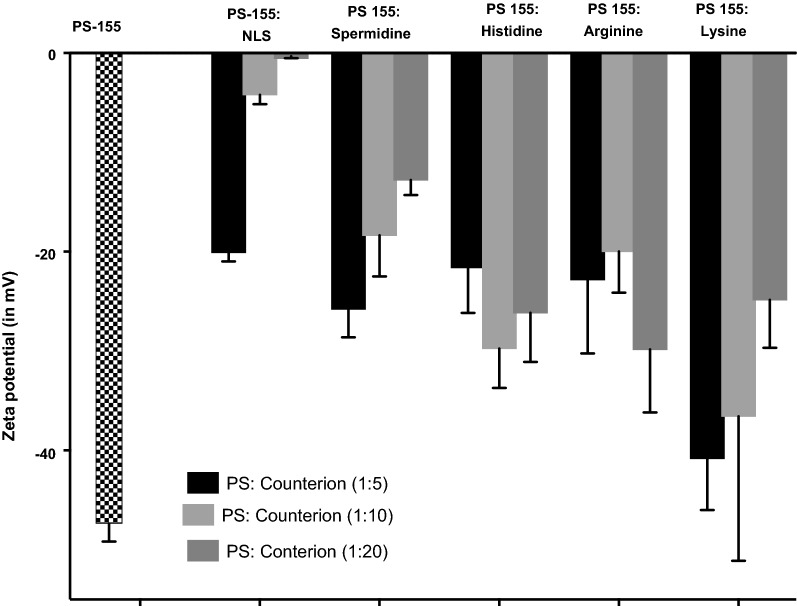
Zeta potential measurement studies. Surface charge (or zeta potential) of encapsulants prepared by mixing different ratios of antimiR-155 PS with indicated counterions. Error bars indicate standard error (± SE) for n = 3

**Fig. 5:**
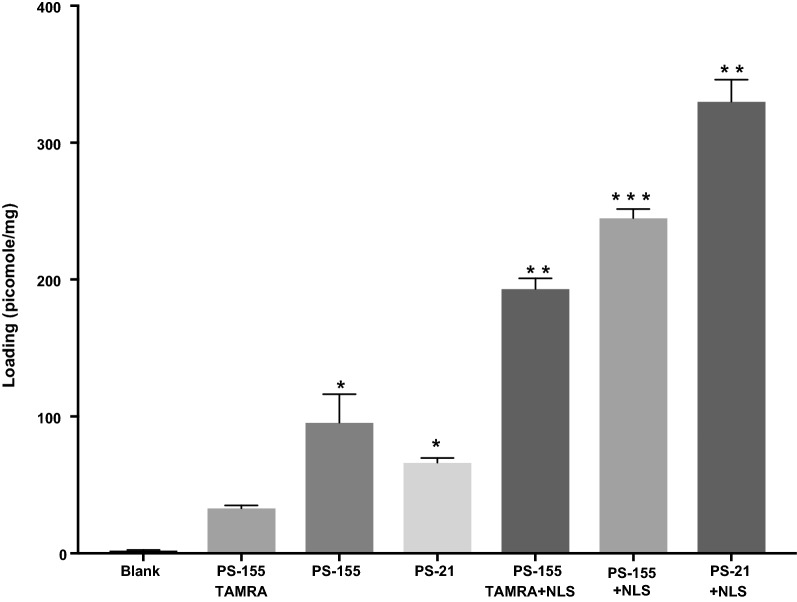
Effect on loading in the presence of NLS. Loading of antimiR PS-155 and antimiR PS-21 with and without NLS in PLGA NPs using absorbance measurement at 260 nm. Data are shown as mean ± standard error (SE), n = 3. *p < 0.05

First, we examined whether complexation between PS-155 and counter-ion decrease the net negative charge of PS-155/counter ion mixture. We mixed PS-155 and counter-ions at three different ratios (1:5, 1:10 and 1:20) and determined the charge density of complexes via zeta potential measurement. We noticed that PS-155/NLS complexes at all three ratios have significantly less negative charge as compared to PS-155 alone as well as complexes containing PS-155 and other counter-ions (Fig. 4). In order to minimize the toxicity caused by positive charge on NP formulation, we pursued only 1:5 ratio of PS-155/NLS complex for subsequent studies. Double solvent evaporation technique was used to encapsulate PS-155/NLS into PLGA NPs. We did not notice any significant decrease in PLGA NPs yield due to NLS during formulation process. We noticed that NLS increased the loading of PS-155 about three-fold higher as compared to PS-155 alone (Fig. 5). To validate our results and confirm whether NLS in general increases the loading of different PS oligonucleotides into NP2, we performed loading studies on NP2 encapsulating PS targeting miR-21 (PS-21) with NLS combination. Consistent with our prior results with PS-155, we noticed that NLS also increased the loading of PS-21 about fourfold higher as compared to PS-21 alone (Fig. 5). In addition, we also noticed that addition of NLS as encapsulant does not affect the NP2 morphology, size as well as surface charge density (Table 2 and Additional file 1: Fig S2). Next, to test whether encapsulation with NLS could affect the release kinetics of PS-155 from NP2 and the binding to target miR-155 sequence, we performed time dependent release study as well as gel shift analysis. Our results confirmed that NLS does not impede the release (Additional file 1: Fig S3) of PS-155 though we noticed difference in release of PS-155 vs PS155-NLS in the first 6 h of release studies. Further, we also confirmed the integrity as well as binding of PS-155 with target miR-155 after in vitro release (Additional file 1: Fig S4). As evidenced by the retarded band, the released PS-155 is bound to the miR-155 target.

**Table 2:** Characterization of PLGA NP2 preparations containing PS-155 and PS-21 alone as well as in combination with NLS for mean size (DLS diameter), surface charge (zeta potential) and polydispersity index (PDI)

PLGA polymer (NP2)	Zeta potential (mv ± SD)	DLS size (nm) ± SD	PDI
PS-155	− 26.5 ± 2.0	376 ± 15	0.16 ± 0.05
PS-155 + NLS	− 23.0 ± 1.3	355 ± 24	0.15 ± 0.04
PS-21	− 32.8 ± 1.8	357 ± 7	0.38 ± 0.04
PS-21 + NLS	− 26.2 ± 2.0	333 ± 9	0.13 ± 0.05

### Cellular uptake studies

**Fig. 6:**
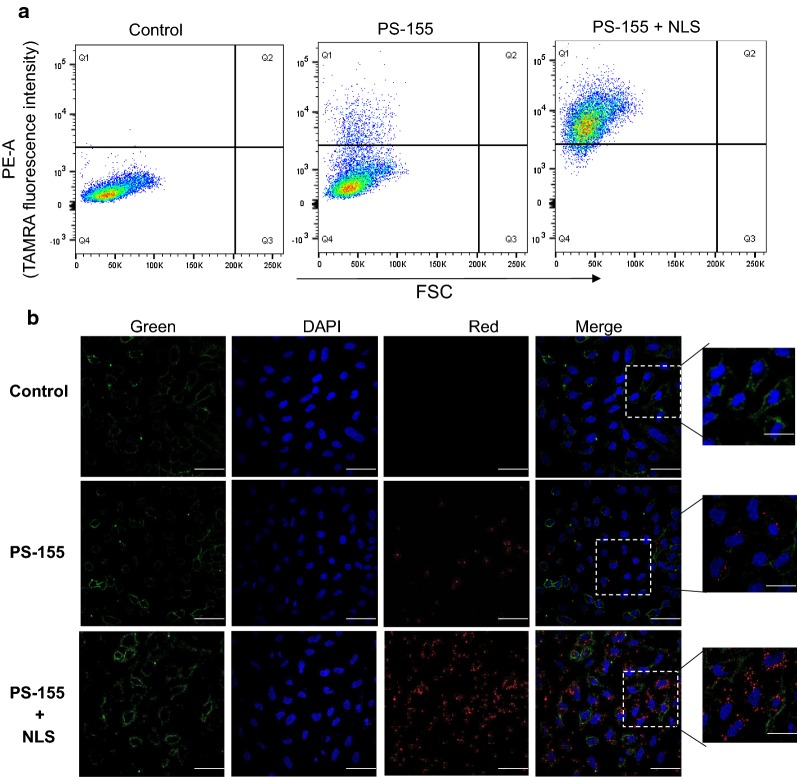
Cellular uptake studies. **a** FACS analysis of A549 cells following incubation with the PLGA NPs containing PS-155 and PS-155/NLS combination (10,000 cells were selected for the number of events). **b** Fluorescent images of A549 cells incubated with PLGA NPs for 24 h, followed by brief washing with PBS and incubation with DAPI (nuclear staining) and MemBrite™ (cell membrane staining). Blue: nucleus (DAPI), Red: PS oligomers (TAMRA), Green (cell membrane). The images were elaborated with ImageJ for clarity purpose

To assess the cellular uptake properties of formulated NPs, we used 5-carboxytetramethylrhodamine (TAMRA) conjugated PS-155 (PS-155-TAMRA). It has been known that loading of antimiR-dye conjugates in NPs is challenging due to the presence of hydrophobic characteristics of dye [26]. We observed that NLS also increased the loading of PS-155-TAMRA into NP2 ~ four folds (Fig. 5). To quantify cellular uptake, we performed flow cytometry analysis. A549 cells were treated with NP2 containing PS-155-TAMRA/NLS and PS-155-TAMRA alone for 24 h followed by flow cytometry analysis. The percentage of cells exhibiting fluorescence signal was significantly higher in cells treated with NP2 containing PS-155-TAMRA/NLS (90%) in comparison to cells treated with NP2 containing PS-155-TAMRA (40%) (Fig. 6a). No auto fluorescence was observed in the un-transfected cells in the TAMRA channel.

Next, we tested the cellular uptake of NP2 containing PS-155-TAMRA/NLS combinations and compared to the cellular uptake of NP2 containing PS-155-TAMRA alone by confocal microscopy. A549 cells in logarithmic phase were cultured in complete media and incubated with 0.5 mg of NPs. After 24 h of incubation, cells were briefly washed three times with buffer saline and stained with nuclear as well as membrane dyes followed by imaging. PS-155-TAMRA/NLS combinations showed increased uptake in comparison to PLGA NP2 containing PS-155-TAMRA alone (Fig. 6b). Though we noticed significant distribution of PS-155-TAMRA in the cytoplasm, we also noted few puncta of TAMRA in the nuclei of cells treated with NP2 containing PS-155-TAMRA/NLS. We presume that observed nuclear uptake could be due to presence of NLS as it is well established that NLS mediates the nuclear import of cargo by binding to their receptors, known as importins [27]. These results altogether indicate that NLS increases the loading of antimiR-155 PS into PLGA NP2 and also increases cellular uptake further validating our results (Additional file 1: Fig S5).

### Quantification of antimiR activity of PS-155 in cell culture

**Fig. 7:**
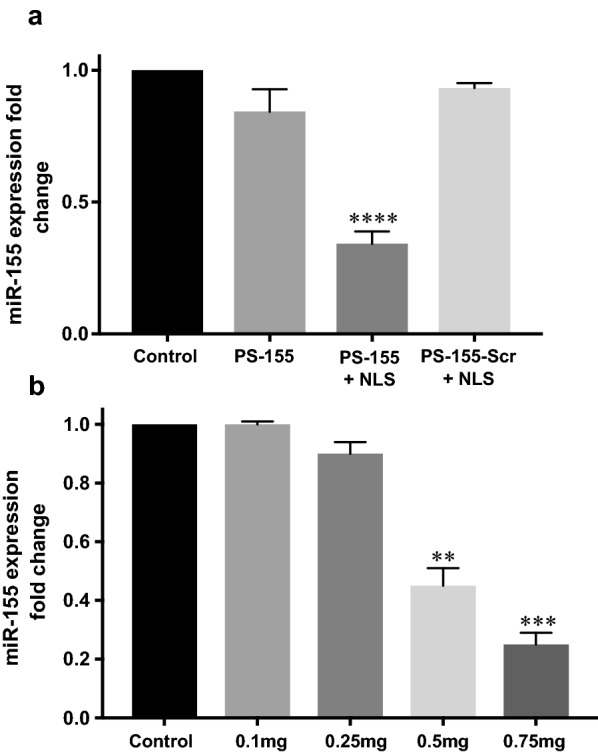
Gene expression analysis results. **a** miR-155 expression in HeLa cells after treatment with PS-155 containing PLGA NPs. miR-155 expression relative to average control (all normalized to U6, n = 3, *p < 0.05). **b** Dose-dependent effect in miR-155 expression level in HeLa cell lines after treatment with different doses of PS-155/NLS containing PLGA NPs (all normalized to U6 control, n = 3, *p < 0.05)

Next, we investigated the antimiR-155 efficacy of formulated NPs by quantitative RT-PCR analysis. For all experiments, PS-155 containing scrambled sequence (PS-155-Scr)/NLS were encapsulated into PLGA NP2 as controls. We tested the expression of miR-155 in both A549 and HeLa cells lines using RT-PCR. HeLa cells exhibited ~ 20-fold higher expression of miR-155 as compared to A549 cells (Additional file 1: Fig S6). Hence, we tested HeLa cell lines for comparing the efficacy of formulated NPs. HeLa cells were treated at a dose of 0.5 mg of PLGA NP2 for 24 h. RNA was extracted and quantified by RT-PCR using U6 as a control. Unlike the scrambled control, PS-155/NLS loaded PLGA NP2 reduced miR-155 expression by ~ 60% (Fig. 7a). Similarly, we also performed a dose–response experiment where HeLa cells were treated with different doses of NP2 containing PS-155/NLS combinations and incubated for 24 h. RT-PCR results demonstrated decreased miR-155 levels as the dose increased (Fig. 7b). In addition, our flow cytometry results also indicate reduction in cell size and an increased cell granularity in NPs containing PS-155/NLS that could be possibly due to its antimiR-155 effect (Additional file 1: Fig S7). We did not notice any change in granularity in case of HeLa cells treated with PS-155-Scr/NLS (Additional file 1: Fig S8). These results signify the antimiR activity of PS-155/NLS loaded PLGA NP2.

### Cell survival studies to test the effect of miR-155 inhibition

**Fig. 8:**
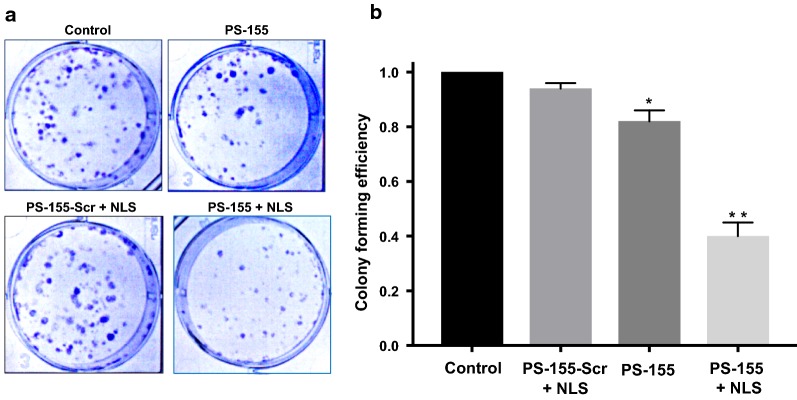
Colonogenic assay results. **a** Representative images and **b** Colony forming efficiency from clonogenic survival assays indicate that pretreatment of cells with PLGA NPs results in a decrease in survival compared to controls. Three independent experiments were performed with n = 3 in each experiment. Hence, data are shown as mean ± standard error (SE). Statistical analysis was performed with Student’s t-test, with *p < 0.05 and **p < 0.005

To assess whether antimiR-155 effect can reduce the survival of HeLa cells, we performed clonogenic assay. HeLa cells were treated with PLGA NPs containing reagents as indicated in Fig. 8. After 24 h, serial dilutions of HeLa cells were performed and ~ 1000 cells from each sample were plated into 6 well plate. After 7 days, staining with crystal violet was performed as indicated in the prior protocol [28]. Cell colonies were counted and imaged for further analysis. We noticed that the cells treated with PS-155/NLS containing NPs showed about 60% reduction in colonies as compared to cells treated with NPs containing PS-155 alone (Fig. 8b).

### Cytotoxicity studies and cytokine array analysis

To assess the safety of PLGA NPs containing PS-155/NLS, we performed cell viability studies using MTT and LDH based assay as well as cytokine array analysis on primary mouse embryonic fibroblast (MEF) cells. Our results indicated that primary MEF cells treated with PLGA NPs containing PS-155/NLS NPs showed no cytotoxicity (Additional file 1: Fig S9) and minimal immune or inflammatory response as compared to control samples (Additional file 1: Fig S10).

## Discussion

Numerous classes of synthetic nucleic acid analogues; PNAs, LNAs and morpholinos delivered via peptides, NPs and liposome based strategies have antimiR effect by sterically blocking the miRNA-mRNA interaction. In contrast, PS oligonucleotides exert their antisense effect targeting mRNA via RNase H-mediated cleavage of target RNA strand in the oligonucleotide-RNA heteroduplex. Most antisense drugs approved by FDA either belong to PS or PS derivatives. Though it has been well established that RNAse H based mechanisms are more potent as compared to steric blockage-based strategies, only few studies report the utility of PS for antimiR-based therapeutics. Overall, major challenge associated with PS-based antimiRs is delivery. Several promising delivery methods; mechanical and/or electrical transduction are investigated to transport PS oligonucleotides [29]. Similarly progress has been made using cationic complexes as well as liposome based formulation [30, 31]. However, despite their promising results, these methods cannot be used for in vivo experiments or therapeutic and diagnostic applications. In addition, stability as well as toxicity passed by cationic formulation remains a big challenge [32]. In this work, we sought to optimize the PLGA-based nano-formulations that can encapsulate surplus amount of PS-based antimiRs with superior transfection efficiency as well as efficacy without exerting any toxic effects. We found that acid terminated PLGA NP2 (21.0–43.5 kDa) efficiently encapsulate antimiR PS-155 with uniform size, morphology and average diameter size of less than 200 nm. Further, NP2 showed improved loading and release of PS-155 as compared to NPs formulated with other grade of PLGA polymers. Though encouraging, there was still only modest encapsulation of PS-155 in PLGA NPs. This could be probably due to interaction of negative charge on acid terminated PLGA polymer with negatively charged PS.

Despite of the modest loading of PS in initial studies, we devised two novel strategies to boost their loading considering their safety profile in prior studies. First, use of cationic delivery vehicles like poly-β-amino ester (PBAE)/PLGA [25] blends or polyethylamine (PEI) [33] and second, use of counter ions to mask negative charge of PS backbone. Many cationic polymers are effective at improving loading of oligonucleotides into NPs. It has been reported that 15% PBAE in PLGA formulations are optimum for loading without causing any adverse effects [25, 34]. However, increasing cationic scaffold (PBAE) beyond this ratio leads to toxicity due to their impact on the cell membranes. Hence, we sought out for methods to reduce the toxicity and increase the payload of PS based antimiRs in PLGA NPs using counter-ions. Our zeta potential results identified that polyplex formed between PS-155 and NLS demonstrates decrease in charge of PS/NLS complex. In addition, zeta potential studies of NPs also depicted that NLS containing formulation does not lead to decrease in the overall negative charge of PLGA formulations (Table 2). These results signify that NLS is encapsulated within the nanoparticles and not loosely bound to its surface. Excitingly, we noticed that NLS dramatically increases the loading of PS-155 into NPs. We also found that NLS increases PS-21 loading onto NPs. Our findings suggest that PS-21 loading was high as compared to PS-155. One plausible explantation could be PS-155 and PS-21 contains different purine and pyrimidine content that contributes to the hydrophilicity of these antimiR oligonucleotides. Though NLS could increase the loading of PS based oligonucleotides but amount of loading of diverse antimiRs can also be incumbent on their purine/pyrimidine content. Our cell culture studies further substantiate that PLGA NPs containing PS-155-TAMRA/NLS show significant uptake in A549 and HeLa cells. Additionally, some PS-155-TAMRA was also confined in the nucleus in case of NLS containing PLGA NPs. These results were consistent with prior studies establishing the role of NLS to traverse molecules across the nuclear membrane. This could also open up new avenues of targeting pre-miRs in the nucleus for more potent antimiR therapy.

Recently, the field of RNA medicine has garnered a lot of interest with the recent successful approval of first RNAi drug Onpattro™ (patisiran) [35] and the clinical success of the RNA targeting oligonucleotide drug Spinraza™ (nusinersen) [36]. Very recently, Akcea and Ionis also received FDA approval for TEGSEDI™ (inotersen) for the treatment of the polyneuropathy of hereditary transthyretin-mediated amyloidosis in adults [37]. However, all these molecules exhibit their action targeting mRNAs only. Some success has been made for targeting miRs using LNAs like miravirsen that has been in clinical trials for targeting miR-122. Recently, clinical trials with LNA based antimiRs have begun for hematopoietic malignancies (Phase 1 Study of cobomarsen (MRG-106) in Cutaneous T cell Lymphoma (CTCL) and HTLV-1 associated T cell Leukemia/Lymphoma) [38, 39]. Though, first and second-generation oligonucleotides have been used massively for targeting mRNA but miRNA targeting has not been explored yet. However, PS based therapies have encountered obstacles in clinical testing mainly due to their delivery challenges.

Results presented in this study are not limited to formulate PLGA NPs encompassing PS but also set a platform for encapsulating second generation antisense oligonucleotides backbone modifications; 2-O-methyl (2-OMe) [40], 2-fluoro (2′-F) [41], 2′-O-methoxyethyl (2′-MOE) [42] sugar substitutions; and LNA [43]; for a number of antimiR-based applications.

## Conclusion

Here, we identified novel PLGA grade polymer to encapsulate PS and further investigated NLS based counter ions to increase the loading as well as transfection efficiency of antimiR-155. In this case, the addition of the NLS to the formulation—provides at least two functions: (1) increased loading of PS-155 within the NPs and (2) increased transfection efficiency of PS-155 in cell-based studies. It is well known that PLGA NPs target the tumor microenvironment by enhanced permeability and retention (EPR) effect, further studies will seek to test the effect of optimized PLGA nano-formulations on oncomiR addicted in vivo tumor models. Specifically, these nano-formulations can be tested on miR-155 transgenic mouse model developed by Slack lab [22] as well as patient derived xenograft models [44]. Overall, this system represents a novel platform for PS delivery with potential therapeutic applications in disease, especially cancer.

## Methods

### Oligonucleotides

PS-155 and PS-21 targeting miR-155 and miR-21 and scrambled PS-155 sequence as well as their TAMRA derivatives were purchased from Midland Certified Reagent (Texas, USA). Following sequences have been used, PS-155; 5′ ACCCCTATCACGATTAGCATTAA 3′, PS-155-Scr; 5′ CTTTTGCATCTAGCACAACACAA 3′ and PS-21; 5′ TCAACATCAGTCTGATAAGCTA 3′.

### Formulation of PLGA NPs

NPs were prepared by double emulsion solvent evaporation method. 80 mg of PLGA polymer was dissolved in 2 mL of DCM overnight. PS-155 or PS-155-Scr were used at 1 nmol/mg of PLGA polymer for loading into NPs. PS was added dropwise to the PLGA solution in DCM followed by ultrasonication (3 × 10 s) resulting in formation of first water in oil (w/o) emulsion. This w/o emulsion was then added slowly into the 5% polyvinyl alcohol (PVA) solution with continuous stirring and ultrasonication (3 × 10 s) to form second water–oil-water emulsion (w/o/w). This double emulsion was then added to 0.3% PVA solution which was stirred overnight allowing DCM to evaporate. NPs obtained were washed three times with ice cold water (4 °C) followed by centrifugation at a speed of 9500 RPM at 4 °C. NPs were suspended in 5 mg/mL of trehalose solution and freeze dried. Lyophilized NPs were stored at − 20 °C.

### Gel shift assays

miR-155 target was incubated with PS-155 or PS-155/NLS in 10 mM sodium phosphate (pH 7.4) at 37 °C in thermal cycler (T100™, Bio-Rad, Hercules, CA). The samples were separated on precasted, 10% nondenaturing polyacrylamide gels using 1× tris/borate/ethylenediaminetetraacetic acid (EDTA) buffer. The gels were run at 120 V for 35 min. After electrophoresis, the gels were stained with SYBR-Gold (Invitrogen) for 3 min and then imaged using a Gel Doc EZ imager (Bio-Rad, Hercules, CA).

### Loading studies

Loading of PS oligonucleotides in PLGA NPs was determined by dispersing  2 mg of NPs in 0.2 mL of DCM at 37 °C. After 3 h, 0.2 mL of Tris/EDTA, pH 7.4 (TE buffer) was added to the DCM, vortexed and agitated for 30 min at a speed of 1000 RPM and at 37 °C. Amount of PS oligonucleotides extracted was measured by centrifuging the samples at 15,000 RPM for 5 min and finally concentration was determined using Nanodrop 8000 (Thermo Scientific, Waltham, MA).

### Release profile studies

Release of PS oligonucleotides from PLGA NPs was analyzed by dispersing 2 mg of NPs in 0.3 mL of phosphate buffer saline (PBS) and agitating on a shaker at a speed of 300 RPM at 37 °C. NPs were centrifuged at 15,000 RPM for 10 min and supernatant was extracted at different time points. Fresh PBS was added after each time point and amount of PS released was measured by determining the absorbance at 260 nm using Nanodrop 8000 (Thermo Scientific, Waltham, MA).

### Scanning electron microscopy images

NPs were sputter coated with palladium for a duration of 2 min. Images were captured using a voltage of 2.0 kV and at 10,000× magnification. Images were further analyzed using ImageJ software (National Institute of Health, Bethesda, MD) to determine the dry size of NPs.

### Characterization of nanoparticles

The hydrodynamic diameter and size distribution of NPs was measured by DLS using non-invasive back scatter technology of Zetasizer Nano ZS (Malvern Panalytical Inc., Westborough, MA, USA). Temperature was kept at 25 °C and refractive index of 1.33 was used for all measurements. Zeta potential was measured using laser doppler micro-electrophoresis technique (Zetasizer Nano ZS, Malvern Panalytical Inc., Westborough, MA, USA) at 25 °C. The Smoluchowski approximation was used. Samples from three different batches were analyzed and average values were reported.

### Cell culture

A549 (ATCC^®^ CCL-185™) and HeLa (ATCC^®^ CCL-2™) cells were obtained from ATCC (Virginia, USA). Cells were cultured in 10 cm petri dishes using dulbecco’s modified eagle medium (DMEM) (Gibco^®^) containing 10% fetal bovine serum (FBS) (Gibco^®^). Cells were passaged when they reached 90% confluency.

### Confocal microscopy

Approximately 100,000 cells (HeLa and A549) were allowed to seed overnight on coverslips in 12 well plates. Cells were treated with 0.5 mg/mL dose of NPs for 24 h. Cells were gently washed with 1 mL PBS (4×) at room temperature (RT). CellBrite™ Fix Membrane Staining dye (1000× dilution) (Biotium, Inc., Fremont, CA, USA) was added followed by incubation at 37 °C for 5 min. Staining dye was aspirated and cells were washed with 1 mL PBS (2×). Cells were fixed by adding 1 mL of 4% paraformaldehyde at 37 °C for 10 min. After washing the cells with 1 mL PBS (2×), cells were permeabilized using 1 mL of 0.1% triton-X in PBS at 37 °C for 10 min. Cells were then washed with PBS (2×) and mounted on a slide using ProLong™ Diamond Antifade Mountant with DAPI (Life Technologies, Carlsbad, CA, USA). Samples were allowed to harden at 4 °C overnight. Samples were imaged using Nikon A1R spectral confocal microscope.

### Flow cytometry analysis

Cells were treated with NPs for 24 h overnight and washed with PBS (4×) followed by trypsinization using 0.25% trypsin–EDTA (Gibco^®^, Life Technologies) at 37 °C for 4 min. Trypsinized cells were suspended in 1 mL of DMEM, 10% FBS media and centrifuged at 1000 RPM for 4 min at 4 °C. After the aspiration of media, cells were washed twice with 1 mL of PBS at 1000 RPM for 4 min at 4 °C. Fixation of cells was done using 300 μL of 4% paraformaldehyde and flow cytometry was done using LSR Fortessa X-20 Cell Analyzer (BD Biosciences, San Jose, CA). Results obtained were analyzed using FlowJo analysis software.

### Real time PCR studies

RNA was extracted from cells using RNeasy Mini Kit (Qiagen, Hilden, Germany). TaqMan™ MicroRNA Assay (Assay ID: 467534_mat) (Applied Biosystem, Foster City, CA) was used to measure miR-155 levels. cDNA was synthesized using miR-155 reverse transcriptase (RT) primers (TaqMan™ MicroRNA Assay), 10× RT buffer, 100 mM dNTPs in presence of RNase inhibitor (Applied Biosystem, Foster City, CA). Reverse transcription was done using the conditions (16 °C for 30 min, 42 °C for 30 min, 85 °C for 5 min) provided with TaqMan™ MicroRNA Assay in thermal cycler (T100™, Bio-Rad, Hercules, CA).

PCR was performed using miR-155 specific primers (TaqMan™ MicroRNA Assay) and TaqMan™ Universal Master Mix II, with UNG (Applied Biosystem, Foster City, CA) using the conditions specified in the assay (50 °C for 2 min, 95 °C for 10 min, (95 °C for 15 s, 60 °C for 60 s) ×40 cycles). U6 snRNA (TaqMan™ microRNA Control) assay was used as control for each sample under similar conditions as miR-155 assay. No template control was used for both control and miR-155 assay. Normalized results relative to control samples were reported.

### Clonogenic assay

Cells treated with 0.5 mg/mL of NPs for 24 h were washed with 1 mL of PBS (4×). After washing, cells were trypsinized using 0.25% trypsin–EDTA (Gibco^®^, Life Technologies) at 37 °C for 4 min. Cells were suspended in 1 mL of DMEM, 10% FBS media and cell count was measured using cell counter (TC20™ Automated Cell Counter, Bio-Rad, Hercules, CA). About 1000 cells from each sample were passaged to 6 well plates and allowed to expand until there were more than 50 cells per colony in untreated sample when viewed under inverted microscope (Laxco™ LMI-3000, Laxco Inc., WA, USA). Colonies were washed with PBS (2×) and fixed using 2 mL of fixation solution (Acetic acid: Methanol, 1:7 v/v) for 5 min at RT. Fixation solution was aspirated and cells were incubated in 1 mL of 0.5% (w/v) crystal violet solution at RT for 2 h. Crystal violet was then removed by immersing wells in tap water and plates were air dried for few days. Number of colonies were counted for each sample and imaged.

### Cell viability assays (MTT and LDH)

Mouse Embryonic Fibroblast (MEF (CF-1) ATCC^®^ SCRC-1040™) cells were purchased from ATCC (Virginia, USA). CellTiter 96^®^ Aqueous One Solution Cell Proliferation assay (Promega, WI, USA) was used to assess the viability of MEF cells after 24 h of treatment with the NPs (PS-155/NLS, PS-155-Scr/NLS and blank). Treated cells were incubated with the reagent for one hour followed by absorption measurement at 490 nm. Samples were prepared in triplicate and cell proliferation data obtained was normalized against control.

Viability of NP treated MEF cells was also determined by measuring the lactate dehydrogenase (LDH) activity using Pierce™ Lactate Dehydrogenase Cytotoxicity Assay Kit (ThermoFisher Scientific, USA). MEF cells were treated with PS-155/NLS, PS-155-Scr/NLS and blank NPs. After 24 h of the treatment, LDH activity was analyzed in the culture medium according to the protocol provided with the kit and measuring the absorbance at 490 nm. Cell lysis buffer was used to obtain the maximum LDH activity. Samples were prepared in triplicate and results were plotted relative to the maximum LDH activity.

## Additional file

**Additional file 1.** Additional figures.

## References

[1] Mishra PJ, Merlino G (2009). MicroRNA reexpression as differentiation therapy in cancer. J Clin Invest.

[2] Slack FJ (2013). MicroRNAs regulate expression of oncogenes. Clin Chem.

[3] Slack FJ, Weidhaas JB (2008). MicroRNA in cancer prognosis. N Engl J Med.

[4] Dias N, Stein CA (2002). Antisense oligonucleotides: basic concepts and mechanisms. Mol Cancer Ther.

[5] Piascik P (1999). Fomiversen sodium approved to treat CMV retinitis. J Am Pharm Assoc.

[6] Wong E, Goldberg T (2014). Mipomersen (kynamro): a novel antisense oligonucleotide inhibitor for the management of homozygous familial hypercholesterolemia. Pharm Ther.

[7] Bonham MA (1995). An assessment of the antisense properties of RNase H-competent and steric-blocking oligomers. Nucleic Acids Res.

[8] Gee JE (1998). Assessment of high-affinity hybridization, RNase H cleavage, and covalent linkage in translation arrest by antisense oligonucleotides. Antisense Nucleic Acid Drug Dev.

[9] Eckstein F (2014). Phosphorothioates, essential components of therapeutic oligonucleotides. Nucleic Acid Ther.

[10] Stenvang J, Petri A, Lindow M, Obad S, Kauppinen S (2012). Inhibition of microRNA function by antimiR oligonucleotides. Silence.

[11] Sanchez-Nino MD, Ortiz A (2013). HCV infection and miravirsen. N Engl J Med.

[12] Agrawal S, Temsamani J, Tang JY (1991). Pharmacokinetics, biodistribution, and stability of oligodeoxynucleotide phosphorothioates in mice. Proc Natl Acad Sci USA.

[13] Clarenc JP, Degols G, Leonetti JP, Milhaud P, Lebleu B (1993). Delivery of antisense oligonucleotides by poly(l-lysine) conjugation and liposome encapsulation. Anticancer Drug Des.

[14] Morishita R (1997). Molecular delivery system for antisense oligonucleotides: enhanced effectiveness of antisense oligonucleotides by HVJ-liposome mediated transfer. J Cardiovasc Pharmacol Ther.

[15] Litzinger DC (1996). Fate of cationic liposomes and their complex with oligonucleotide in vivo. Biochim Biophys Acta.

[16] Ando S, Putnam D, Pack DW, Langer R (1999). PLGA microspheres containing plasmid DNA: preservation of supercoiled DNA via cryo preparation and carbohydrate stabilization. J Pharm Sci.

[17] Cun D (2011). High loading efficiency and sustained release of siRNA encapsulated in PLGA nanoparticles: quality by design optimization and characterization. Eur J Pharm Biopharm.

[18] Gupta A, Bahal R, Gupta M, Glazer PM, Saltzman WM (2016). Nanotechnology for delivery of peptide nucleic acids (PNAs). J Control Release.

[19] Seo YE (2019). Nanoparticle-mediated intratumoral inhibition of miR-21 for improved survival in glioblastoma. Biomaterials.

[20] Malik S, Oyaghire S, Bahal R (2019). Applications of PNA-laden nanoparticles for hematological disorders. Cell Mol Life Sci.

[21] Malik S, Asmara B, Moscato Z, Mukker JK, Bahal R (2019). Advances in nanoparticle-based delivery of next generation peptide nucleic acids. Curr Pharm Des..

[22] Babar IA (2012). Nanoparticle-based therapy in an in vivo microRNA-155 (miR-155)-dependent mouse model of lymphoma. Proc Natl Acad Sci USA.

[23] Gupta A, Bahal R, Gupta M, Glazer PM, Saltzman WM (2016). Nanotechnology for delivery of peptide nucleic acids (PNAs). J Control Release..

[24] Quijano E, Bahal R, Ricciardi A, Saltzman WM, Glazer PM (2017). Therapeutic peptide nucleic acids: principles, limitations, and opportunities. Yale J Biol Med.

[25] Fields RJ (2012). Surface modified poly(β amino ester)-containing nanoparticles for plasmid DNA delivery. J. Control Release.

[26] Zhu H, McShane MJ (2005). Loading of hydrophobic materials into polymer particles: implications for fluorescent nanosensors and drug delivery. J Am Chem Soc.

[27] Fanara P, Hodel MR, Corbett AH, Hodel AE (2000). Quantitative analysis of nuclear localization signal (NLS)-importin alpha interaction through fluorescence depolarization. Evidence for auto-inhibitory regulation of NLS binding. J Biol Chem..

[28] Franken NA, Rodermond HM, Stap J, Haveman J, van Bree C (2006). Clonogenic assay of cells in vitro. Nat Protoc.

[29] Guo Z (2014). Enhanced antisense oligonucleotide delivery using cationic liposomes incorporating fatty acid-modified polyethylenimine. Curr Pharm Biotechnol.

[30] Kanamaru T, Takagi T, Takakura Y, Hashida M (1998). Biological effects and cellular uptake of c-myc antisense oligonucleotides and their cationic liposome complexes. J Drug Target.

[31] Chakraborty R, Dasgupta D, Adhya S, Basu MK (1999). Cationic liposome-encapsulated antisense oligonucleotide mediates efficient killing of intracellular leishmania. Biochem J.

[32] Gokhale PC (2002). Pharmacokinetics, toxicity, and efficacy of ends-modified raf antisense oligodeoxyribonucleotide encapsulated in a novel cationic liposome. Clin Cancer Res.

[33] Kleemann E (2005). Nano-carriers for DNA delivery to the lung based upon a TAT-derived peptide covalently coupled to PEG-PEI. J Control Release.

[34] Fields RJ (2014). Modified poly(lactic-co-glycolic Acid) nanoparticles for enhanced cellular uptake and gene editing in the lung. Adv Healthc Mater.

[35] Garber K (2018). Alnylam launches era of RNAi drugs. Nat Biotechnol.

[36] Corey DR (2017). Nusinersen, an antisense oligonucleotide drug for spinal muscular atrophy. Nat Neurosci.

[37] Benson MD (2018). Inotersen treatment for patients with hereditary transthyretin amyloidosis. N Engl J Med.

[38] Fava P (2017). miR-155 expression in primary cutaneous T-cell lymphomas (CTCL). J Eur Acad Dermatol Venereol.

[39] Seto AG (2018). Cobomarsen, an oligonucleotide inhibitor of miR-155, co-ordinately regulates multiple survival pathways to reduce cellular proliferation and survival in cutaneous T-cell lymphoma. Br J Haematol.

[40] Yoo BH, Bochkareva E, Bochkarev A, Mou TC, Gray DM (2004). 2′-O-methyl-modified phosphorothioate antisense oligonucleotides have reduced non-specific effects in vitro. Nucleic Acids Res.

[41] Kawasaki AM (1993). Uniformly modified 2′-deoxy-2′-fluoro phosphorothioate oligonucleotides as nuclease-resistant antisense compounds with high affinity and specificity for RNA targets. J Med Chem.

[42] Sazani P, Astriab-Fischer A, Kole R (2003). Effects of base modifications on antisense properties of 2′-O-methoxyethyl and PNA oligonucleotides. Antisense Nucleic Acid Drug Dev.

[43] Obad S (2011). Silencing of microRNA families by seed-targeting tiny LNAs. Nat Genet.

[44] Zhang Y (2012). LNA-mediated anti-miR-155 silencing in low-grade B-cell lymphomas. Blood.

