# Supplementary material for: Investigation of PLGA nanoparticles in conjunction with nuclear localization sequence for enhanced delivery of antimiR phosphorothioates in cancer cells in vitro
Source: J Nanobiotechnology. 2019 Apr 22;17:57. doi: 10.1186/s12951-019-0490-2 (PMC6475967; doi:10.1186/s12951-019-0490-2)
Supplement: Supplementary file 1 — Additional file 1. Additional figures. [file 12951_2019_490_MOESM1_ESM.docx]

**Supporting information**

**Investigation of PLGA nanoparticles in conjunction with nuclear localization sequence**

**for enhanced delivery of antimiR phosphorothioates in cancer cells *in vitro***

Shipra Malik^1^, Raman Bahal^1*^

**Fig S1.** Representative size distribution and scanning electron micrographs (SEM) (inset) of PLGA nanoparticles containing PS-155. Bar represents 1 μm. Average particle diameter and SD are given for each nanoparticle batch.

**Fig S2.** Representative size distribution and scanning electron micrographs (SEM) (inset) of PLGA nanoparticles containing PS-155/NLS combination. Bar represents 1 μm. Average particle diameter and SD are given for nanoparticle batch.

**Fig S3.** Cumulative release profile data of antimiR PS-155/NLS from PLGA NP2 at indicated time points in a graph. Data are shown as mean ± s.e.m., n=3.

**Fig S4.** (A**)** Schematic showing work plan to evaluate the release and assessing the *in vitro* binding affinity of PS-155 with target miR-155 **(B)** Gel-shift assay following incubation of miR-155 target with PS-155 and PS-155/NLS in simulated physiological salt conditions at 1:1 ratio **(C)** PS-155 released from PLGA NPs are incubated with miR-155 (1.0uM) and run on PAGE followed by SYBR gold staining.

**Fig S5. (A)** FACS analysis of HeLa cells following incubation with the PLGA NPs containing PS-155 and PS-155/NLS combination (5 000 cells were taken for the events). (B) Fluorescent images of HeLa cells incubated with PLGA NPs for 24 h, followed by brief washing with PBS and incubation with DAPI (Nuclear staining). Blue: nucleus (DAPI), red: PS oligomers(TAMRA).

**S6**

**Fig S6.** Normalized miR-155 expression levels in A549 and HeLa cells using RT-PCR. Data are shown as mean ± s.e.m., n=3.

**S7**

**Fig S7.** FACS contour plots generated from HeLa cells treated with PS-155 and PS-155/NLS containing PLGA NPs.

**S8**

**Fig S8.** FACS contour plots generated from HeLa cells treated with PS-155-Scr/NLS containing PLGA NP2.

**Fig S9**. Safety profile studies on primary MEF cells. (A) MTT and (B) LDH assays were performed onto MEF cells treated with NPs as indicated on x axis at 24 h post treatment. Control include untreated and Blank NP treated cells. Triplicate samples were used (n=3) and data are shown as mean ± s.e.m.

**S10**

**Fig S10.** Analysis of cytokine levels, as indicated, in supernatant collected from MEF cells either untreated or PS-155/NLS NP treated at 24 h post treatment. Data are shown as mean ± s.e.m., n=3.
